# Show Me the Evidence: COVID-19 and School Nursing in the 21st Century

**DOI:** 10.1177/1942602X20974770

**Published:** 2020-12-15

**Authors:** Erin D. Maughan, Kathleen H. Johnson, Juanita Gryfinski, Wendy Lamparelli, Shaylene Chatham, Jeana Lopez-Carrasco

**Affiliations:** National Association of School Nurses, Silver Spring, MD; University of Washington, Seattle, WA; St. Charles Community Unit School District 303, St. Charles, IL; Hackensack Public School District, Hackensack, NJ; Mingo Valley Christian, Tulsa, OK; Nashua School District, Nashua, NH

**Keywords:** evidence-based practice, COVID-19, *Framework for 21st Century School Nursing PracticeTM*, Standards of Practice, school nursing advocacy

## Abstract

The emergence of COVID-19 and how to control its spread has highlighted the importance of understanding and applying evidence-based decisions into school nursing practice. This is the fifth and final article in NASN’s series on how the Framework for 21st Century School Nursing PracticeTM is a mind-set that can be applied to everyday school nursing practice and will focus on the principle of Standards of Practice, and particularly how evidence-based practice decisions are made during COVID-19.

**Figure:**
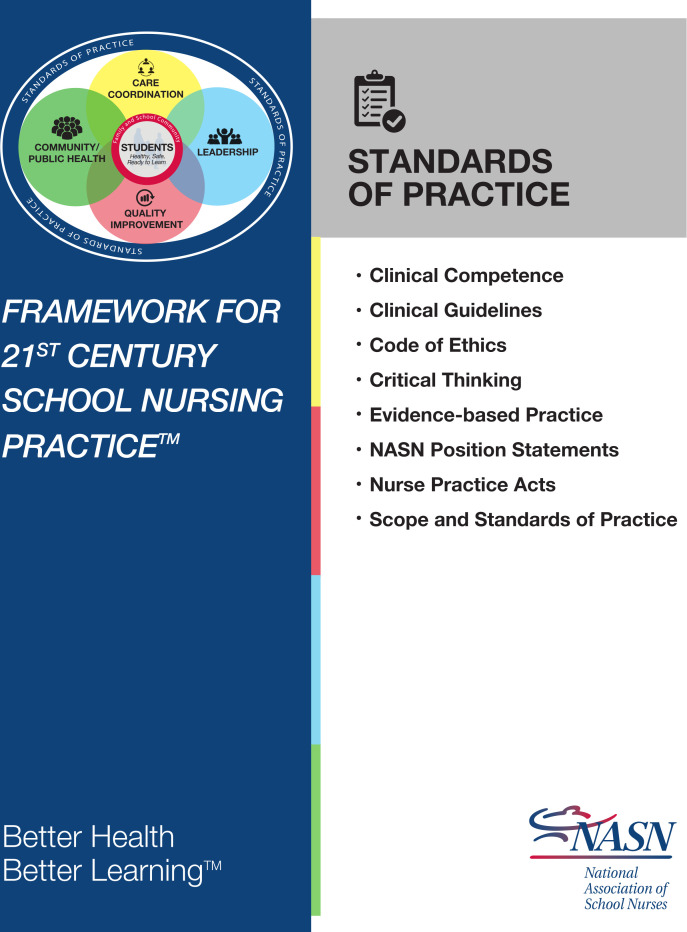


Should students wear masks in schools? Is it safe for schools to meet in person? Is the wrist an appropriate site for temperature taking? What does the current evidence say? These are some of the questions school nurses have been asked as schools cope with the implications of coronavirus disease 2019 (COVID-19). The emergence and continuing evolution of COVID-19 has highlighted the importance of understanding and applying evidence-based decisions into school nursing practice.

Evidence-based practice is not just using information that has a reference. It includes the best available research and scientific evidence, as well as data from school nurse documentation. In addition, evidence-based practice decisions must be made based on the availability of resources and clinical expertise, and accounts for the culture and needs of the targeted population (Melnyk & Fineout-Overholt, 2015; National Association of School Nurses [NASN], 2020). A Venn diagram helps illustrate that evidence-based practice is the intersection of evidence, resources, and a population’s culture (Figure 1) (Maughan & Yonkaitis, 2017).

**Figure 1.**
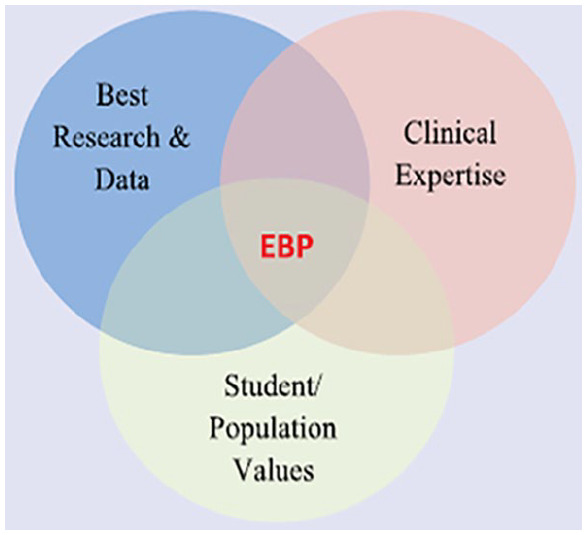
Evidence-Based Practice (EBP)

Evidence-based practice is a practice component in the principle of Standards of Practice in NASN’s *Framework for 21st Century School Nursing PracticeTM (Framework)*. The *Framework* consists of five nonhierarchical principles. The principle Standards of Practice is depicted surrounding the other four principles, because it underlies all actions and thoughts of a school nurse (NASN, 2020). The *Framework* was developed to help guide school nursing practice by creating a mindset for school nurses to embrace (NASN, 2016, 2020). This is the final article in NASN’s series on how the *Framework* mind-set and principles can be applied to everyday school nursing practice. This article focuses on the principle of Standards of Practice, particularly evidence-based practice decisions, using COVID-19 as the example.

NASN staff reached out to several nurses across the country who indicated they were involved in creating state or district level COVID-19 policies. We asked the nurses if they would share how they used evidence and stayed current during a complex time of rapidly evolving evidence. The nurses who responded are authors of this article. They are referred to in third person because many also shared experiences of multiple nurses in their state to represent a broader community. Collectively, their answers illustrated the key components of evidence-based practice (Figure 1).

## Best, Current Evidence

**Figure 2.**
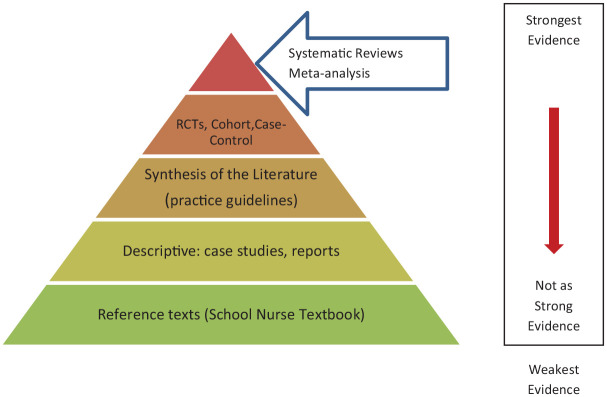
Pyramid of Evidence *Note*. RCT = randomized controlled trials.

Evidence comes from many sources. Traditionally, peer-reviewed research articles discussing systematic reviews of multiple studies are considered the gold standard, and strongest evidence (Melnyk & Fineout-Overholt, 2015). See Figure 2. Those school nurse authors who had access to the journals indicated they searched and read all the applicable medical and public health articles. They even read articles published about schools reopening in Europe, in hopes of gleaning lessons learned. However, many school nurses do not have access to online research articles, which makes it hard to stay current. The nurse authors also looked for the strongest evidence in the literature that came from reputable organizations (i.e., “.gov” or “.edu”) that was peer reviewed or disseminated by trusted experts. Due to the evolving nature of COVID-19, much of the current evidence was not always published in the journals yet. The nurse authors also relied on other research studies, as well as experts such as the Centers of Disease Control and Prevention (CDC), the National Institute of Occupational Safety and Health, and the World Health Organization who compile various sources of evidence (Maughan & Yonkaitis, 2018; Yonkaitis, & Maughan, 2018b).

COVID-19 evolved so quickly that it was challenging to track current evidence. While most studies are not released before they are peer reviewed by other experts, because COVID-19 continues to evolve and has such urgent implications for saving lives, many studies were released before the peer review process. Other data were analyzed quickly and not always published in journals. This made finding the strongest evidence difficult for many school nurses. In these situations, a review of multiple credible sources, together with input from experts such as the CDC or state epidemiologists, is appropriate. For example, the CDC (2020a, 2020b, 2020c) provided guidance on students wearing masks, opening schools, and infection control policies. The information was based on the best evidence available at the time, as decided upon by multiple experts. In this case, the combination of sources provided the best evidence.

Just like the CDC has access to the primary evidence, state level of government employees may have access to the primary literature and have connections with state experts. The nurse authors all mentioned state sources who helped provide synopses and guidance. Approximately 32 states employ a state school nurse consultant in either their state health or state education department (S. Trefry, personal communication, October 2, 2019). These nurses have expertise in school nursing and are an invaluable resource for local school nurses providing consistent information for all schools. For example, a state school nurse consultant, using resources from the CDC, Occupational Safety and Health Administration (OSHA), and American Nurses Association, developed a system to identify and deliver appropriate personal protective equipment supply kits to all the schools in North Carolina (A. Nichols to S. Trefry, personal communication, June 13, 2020).

State school nurse consultants also proactively developed materials to help guide school nurses and answer questions as evidence evolved about COVID-19. For example, concern was raised about the accuracy of infrared forehead thermometers when the weather turned colder and students were in heated cars. Some school nurses found reports that indicated checking temperatures on the wrist provided the same results. State school nurse consultants were able to direct school nurses to the strong research evidence, which indicated temperature taking on the wrist was not accurate (Chen et al., 2020). State consultants also work together across states to share information and stay up to date. For example, several consultants worked together to draft guidance for healthcare professionals in the schools related to personal protective equipment, which was reviewed, developed, and finalized with representatives from the NASN (Trefry et al., 2020).

Many states began doing weekly calls with state experts and school nurses. The experts included the state school nurse consultant, state epidemiologist, infectious disease specialists, and even a nurse practitioner who guided the nurses through various scenarios, so they knew what to do. In states with a consultant, the state school nurse consultant often organized the calls. The calls allowed evidence and resources to be shared, school nurses to be informed of efforts in their own states, and schools nurses to share their own data, outcomes, and observations from their specific population.

School nurses’ own data are another source of evidence for local decisions and evidence of evolving trends. Data points specific to COVID-19 have been identified to provide guidance to school nurses (Maughan & Bergren, 2020). Several of the nurse authors thought early which data points to track and use to tell their story. The *National School Health Database: Every Student Counts!* provides the infrastructure for school nurses to use the data in their own documentation as evidence of what is happening with their own population of students and families (NASN, n.d.). Compiling these sources of data together provide schools nurses with the best evidence.

## Clinical Expertise and Application

Evidence-based decisions do not just include what the best evidence shows but must address what resources and clinical expertise is available, and how the data fits with the population’s own preferences (Melnyk & Fineout-Overholt, 2015; Yonkaitis & Maughan, 2018a). This was demonstrated as several school nurses participated in state- and/or district-level COVID-19 teams. These teams were often made up of state school nurse consultants, school administrators, workforce specialists, school nurses, epidemiologists, infectious disease, and other health experts who helped provide guidance. Having a team allowed the group to share the work and address limited resources in the state. State Nurse Practice Acts, another practice component in the Standards of Practice principle of the *Framework*, and other laws and guidelines had to be considered as they affected how a nurse works within full scope of practice (American Nurses Association & NASN, 2017; NASN, 2020). These are all examples of the evidence-based decision-making process.

Often, state policies were brought to the district level, where teams of school nurses and other experts adapted the plans to their district. For example, a district with one nurse covering multiple schools could not carry out the plan the same way as a district with a nurse in each school. Critical thinking, another practice component in Standards of Practice, was utilized by school nurses during each of these steps when applying the evidence to the current situation in their district (related to staffing, resources, and cultural needs).

## Tips and Suggestions

The nurse authors shared tips or advice to help others, knowing that not all school nurses were having positive experiences in their schools. The uncertain and evolving nature of COVID-19 evidence has made the process of providing recommendations and implementing evidence-based practice more stressful. In these situations, the nurses reminded themselves and school administrators this was the most current evidence and that it may change as more was learned. Expectations for the fluid nature of the situation were set early. Nurses spoke to the importance of self-awareness and emotional intelligence in understanding themselves in situations to help modulate their own responses to their work on COVID-19.

The nurse authors also indicated that they had spent years working with educators to build a relationship of trust and show their credibility. They had worked hard to stay up to date and use evidence (including the provision of references on documents) when speaking with decision-makers and developing policies, not just during a crisis. This investment paid off during this current crisis because the relationship of trust was in place.

Many school nurses also indicated they learned to be proactive and invite themselves to sit and stay at the table providing examples from their own data. They networked and shared what they knew. This led some of them to speak at town halls and at school board meetings. Another common observation was that they started their data collection and analysis early. They identified what data would help guide their practice, and what data would help them tell their story. All of the nurse authors mentioned they spent much of their summer learning, preparing, and communicating with their education leaders because they knew that decisions needed to be made before the fall. This helped educators stay informed and increased the school nurses’ credibility.

Not everything went as planned. Many of the authors spoke to having to tweak and adapt plans and constantly communicate with educators who still asked for decisions to be made that may not have been based on the best evidence. Each situation was different, and a constant struggle but those who stayed current and proactive seemed to have the most positive experiences.

## Mind-set

The year 2020 and COVID-19 has brought new challenges to school nurses in how they carry out their work. The use of current evidence has been critical in keeping students safe, whether they were learning virtually or in person. In addition to using evidence, COVID-19 has highlighted the need for a professional and proactive mindset, which is highlighted in NASN’s *Framework* (NASN, 2016, 2020). This article focused on the principle of Standards of Practice; however, we can see examples of the other principles working together to form this mindset. School nurses used practice components found in the Leadership principle as they advocated for their students’ needs, created new policies, and used new technology. Their team approach was often interdisciplinary and required collaborative communication with multiple entities that are practice components in the principle of Care Coordination. Using the principle of Community/Public Health, school nurses were the front-line workers in the control of COVID-19 through their work in tracking cases (surveillance), identifying students at risk (risk reduction), and teaching the school community regarding COVID-19 (health education). Finally, nurses used practice components in the principle of Quality Improvement when they applied their plans to practice, collected data, and evaluated how well the plans worked (NASN, 2016, 2020). Using the *Framework* has supported a coordinated professional response by school nurses in their response to COVID-19.

## Conclusion

The COVID-19 crisis is not over, the examples of evidence-based practice shared by school nurses in this article can still be applied now. Maintain your focus on keeping the school community (including yourself) healthy. Be proactive, stay current and credible; find your team! Together, as school nurses follow the evidence, they can keep students healthy, safe, and ready to learn. ■
